# Supplementary material for: The Plasmodium falciparum cytoplasmic translation apparatus: a promising therapeutic target not yet exploited by clinically approved anti-malarials
Source: Malar J. 2018 Dec 12;17:465. doi: 10.1186/s12936-018-2616-7 (PMC6292128; doi:10.1186/s12936-018-2616-7)
Supplement: Supplementary file 1 — Additional file 1. Method for preparation and calibration of saponin. [file 12936_2018_2616_MOESM1_ESM.docx]

**Additional File 1. Method for preparation and calibration of saponin**

Because Saponin Quillaja (Sigma #S4521) is a natural product isolated from tree bark, preparations are very heterogeneous, with content of the active lysing agent, sapogenin, ranging from 20-35%, and considerable variation in potency even within lots. We therefore prepare large batches (10 grams) of saponin at 0.15% w/v in phosphate buffered saline. This preparation is subjected to dual-stage filtration: first filtered with 0.8μm nitrocellulose filtration to remove large particulate, then 0.2μm nitrocellulose sterile filtration. Filtered saponin solution is then frozen and stored in 50mL aliquots at -20°C, prior to testing/calibration.

Each batch is calibrated empirically in a pairwise manner with test harvests to determine the optimum amount of aliquoted saponin stock to be used. For calibration of saponin, parasites should be cultured at 2% hematocrit and 15-20% parasitemia. Each test harvest consists of 1 liter of culture in 2 HYPERFlasks. HYPERFlasks should be mixed to resuspend settled RBCs/iRBCs, and then carefully poured into sterile 500mL polypropylene centrifuge tubes. Tubes are centrifuged at 1500rpm in a tabletop centrifuge for 5 minutes at room temperature without brake. Media is then carefully removed from the RBCs/iRBCs. At this point, RBCs/iRBCs from both tubes should be resuspended in a small amount of ice cold Buffer A (20mM HEPES pH8.0, 2mM Mg(OAc)_2_, 120mM KOAc) and mixed together to create one pool of culture. This culture should then be evenly divided between 2x50mL conical-bottom tubes (tubes should be rated for high speeds above 10,000x*g*) and placed on ice. The volume in one tube (Tube 1) should be brought up to 38mL with ice-cold Buffer A, and the volume in the other tube (Tube 2) should be brought up to 40mL with ice-cold Buffer A. Ice-cold saponin, previously thawed at room temperature and then placed on ice, should be added as quickly as possible to the tubes to bring the volume up to 50mL in each tube: 12mL saponin to Tube 1 and 10mL saponin to Tube 2. The tubes should be quickly inverted 2-3 times (first making sure the caps are properly threaded and screwed on tightly), returned to ice, and then centrifuged immediately at 10,000x*g* at 4^o^C for 10 minutes with low brake. Keeping tubes on ice, carefully but quickly remove supernatant, then resuspend in each pellet in 45mL ice-cold Buffer A to wash, and then repeat centrifugation at 10,000x*g.* Repeat wash and centrifugation once again. After wash, carefully remove supernatant, and resuspend each pellet in an equal volume of Buffer B2 (20mM HEPES pH 8.0, 100mM KOAc, 0.75mM Mg(OAC)_2_, 2mM DTT, 20% glycerol, 1X cOmplete EDTA-free protease inhibitor cocktail (Roche #4693132001)). For instance, if parasite pellet after washes is 1mL in volume, add 1mL of Buffer B2 for a total of 2mL volume. Transfer the parasites resuspended in Buffer B2 to separate 1.5mL screw-top tubes, flash-freeze in liquid nitrogen, and store at -80^o^C. Process the pellets according to the protocol detailed below, then test each in the PfIVT assay as detailed below across a range of added magnesium (2.0, 2.5, 3.0, 3.5mM final concentration added with 10XTM). Whichever saponin amount yielded the extract with the highest signal, utilize that amount in the next pairwise test, following the flow chart in Additional File 2 and repeating testing until optimum concentration determined. For instance, if 10mL of saponin yields the more active extract in the test Round 1, 10mL and 8mL will be utilized in test Round 2, and so on, according to the flowchart (Additional File 2). This will require 3 to 5 rounds of harvesting and pairwise testing to determine the appropriate amount of saponin to use from each large batch of saponin solution generated. Utilize this empirically-determined amount of saponin for all lyses conducted with aliquots from that batch.
