# Supplementary figures and images for: The Plasmodium falciparum cytoplasmic translation apparatus: a promising therapeutic target not yet exploited by clinically approved anti-malarials
Source: Malar J. 2018 Dec 12;17:465. doi: 10.1186/s12936-018-2616-7 (PMC6292128; doi:10.1186/s12936-018-2616-7)

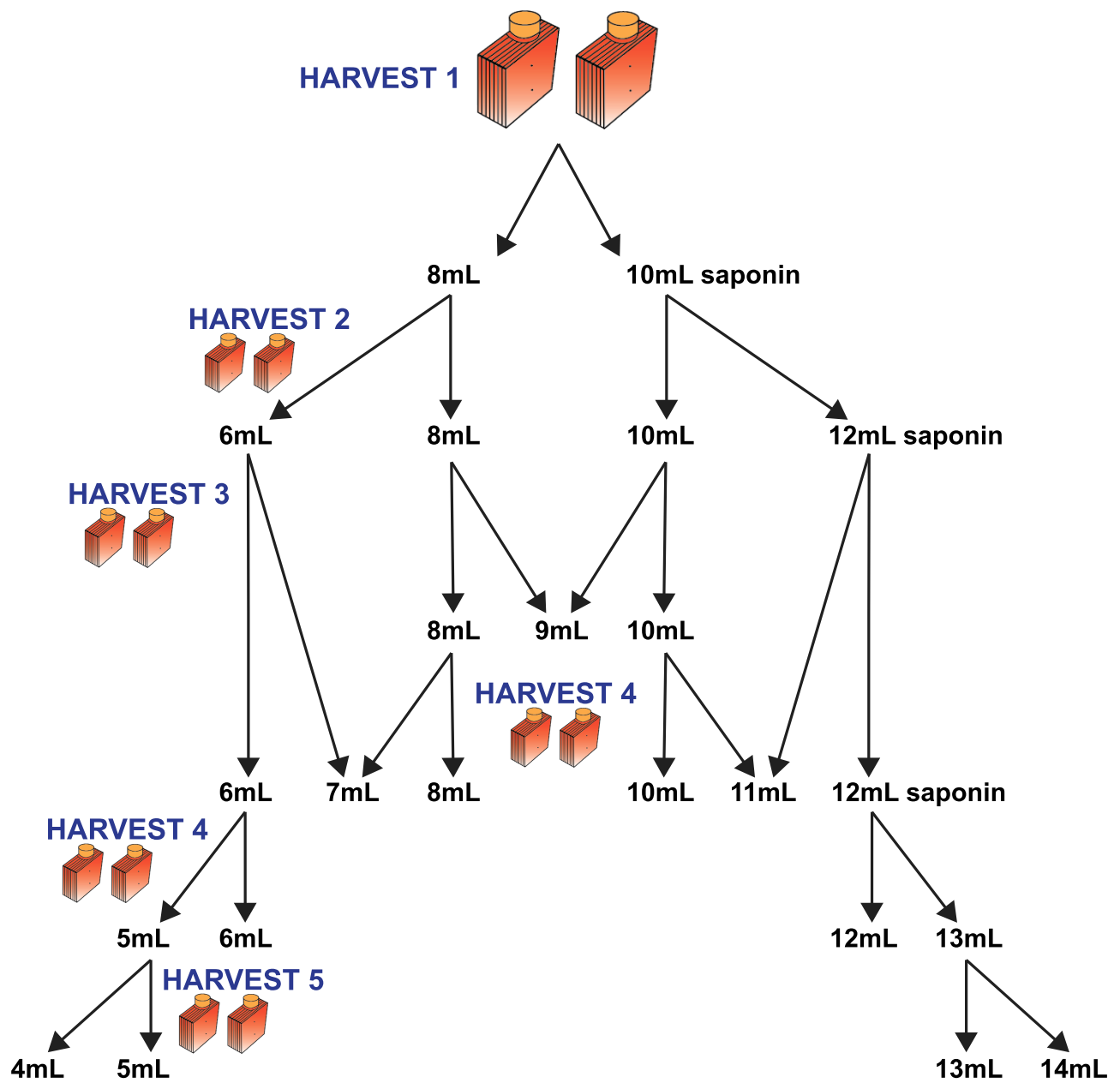

Supplement: Supplementary file 2 — Additional file 2. Flowchart of saponin batch calibration for erythrocyte lysis. Saponin amounts for RBC lysis are empirically determined through pairwise comparison for each preparation/batch of saponin. 3 to 5 harvests and pairwise tests will be required to determine the ideal amount of saponin for a given batch. Volumes indicated on the flowchart are for the volume of 0.15% saponin (in Buffer A) to be added to parasites in Buffer A and to a total volume of 50 mL. Harvest 1 should be tested with 8 mL and 10 mL saponin. Subsequent pairs for testing are determined by following the arrows on the flowchart: if 8 mL yields the more active extract in Harvest 1, Harvest 2 will compare 8 mL with 6 mL saponin; if 6 mL yields the more active extract in Harvest 2, Harvest 3 will compare 6 mL with 7 mL saponin, and so on, until a final value has been reached. [file 12936_2018_2616_MOESM2_ESM.pdf]

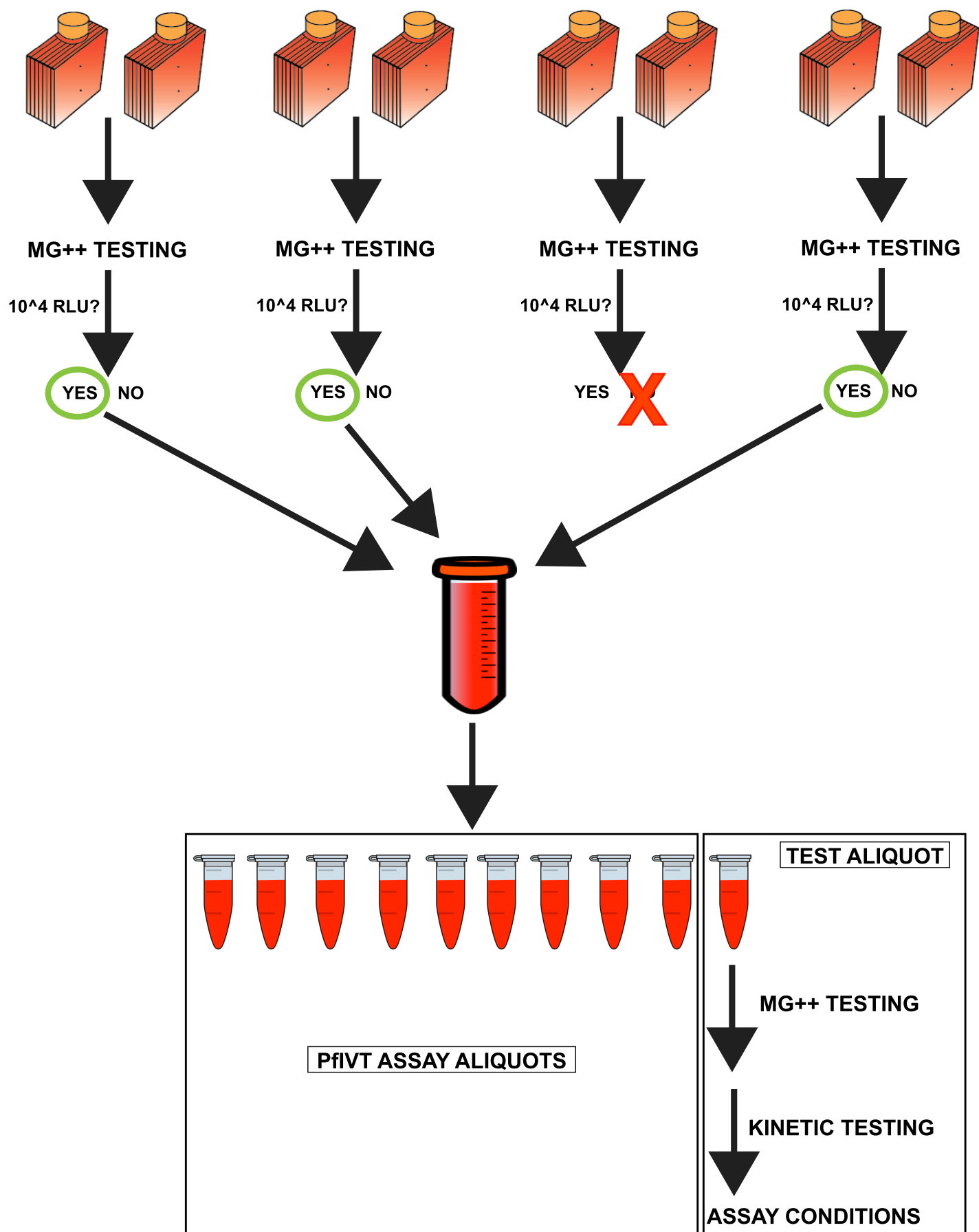

Supplement: Supplementary file 4 — Additional file 4. Flowchart for PfIVT extract quality control and pooling. Individual harvests are first tested at different magnesium concentrations; those that achieve 104 RLU activity threshold are pooled. Pooled extract is aliquoted, and a test aliquot is utilized to first determine ideal magnesium concentration, then ideal incubation time. Remaining aliquots are utilized for PfIVT assays at the determined magnesium & kinetic conditions. [file 12936_2018_2616_MOESM4_ESM.pdf]

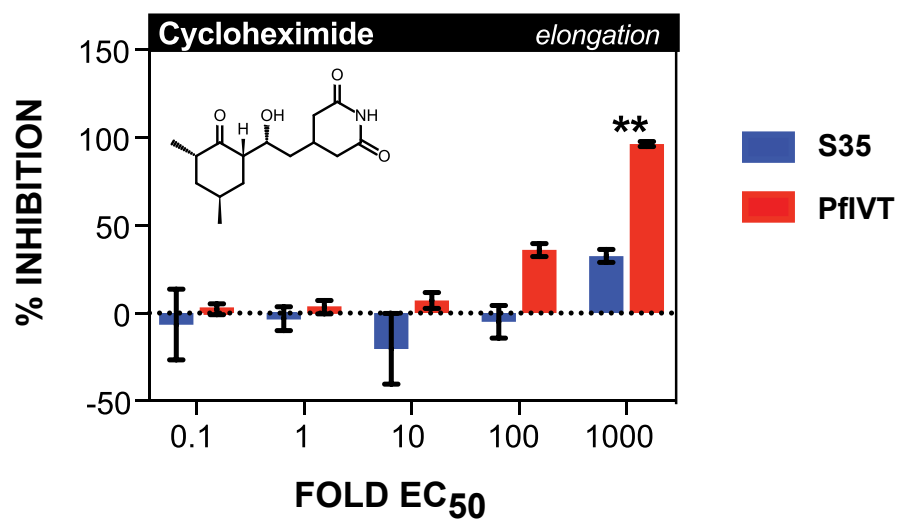

Supplement: Supplementary file 6 — Additional file 6. Dose-dependent inhibition of S35 incorporation and PfIVT assays by cycloheximide. Dose-dependent inhibition of S35 incorporation (blue bars) and PfIVT assays (red bars) by the translation inhibitor cycloheximide, tested up to 1000-fold (**) the EC50 calculated in P. falciparum growth inhibition assay. [file 12936_2018_2616_MOESM6_ESM.pdf]

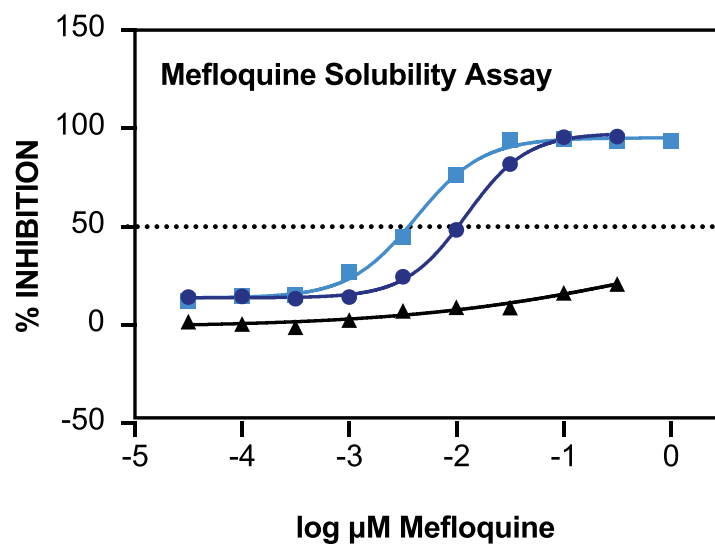

- Mefloquine IVT Extract  $\text{EC}_{50} = 12.31\text{nM}$
- Mefloquine No Extract  $\text{EC}_{50} = 4.17\text{nM}$
- ▲ Extract + DMSO

Supplement: Supplementary file 7 — Additional file 7. Mefloquine solubility assay. Dose-dependent inhibition of P. falciparum in vivo growth by mefloquine in PfIVT extract post-PfIVT reaction (Mefloquine IVT Extract), mefloquine alone (Mefloquine No Extract), or DMSO control in PfIVT extract post-PfIVT reaction (Extract + DMSO). [file 12936_2018_2616_MOESM7_ESM.pdf]
