# Supplementary material for: The Plasmodium falciparum cytoplasmic translation apparatus: a promising therapeutic target not yet exploited by clinically approved anti-malarials
Source: Malar J. 2018 Dec 12;17:465. doi: 10.1186/s12936-018-2616-7 (PMC6292128; doi:10.1186/s12936-018-2616-7)
Supplement: Supplementary file 3 — Additional file 3. Method for culturing and extract generation: step-by-step protocol. [file 12936_2018_2616_MOESM3_ESM.docx]

**Additional File 3. Method for culturing and extract generation: step-by-step protocol.**

1. Double-synchronize parasites with either a) two sorbitol synchronizations or b) 1 sorbitol synchronization and 1 MACS purification
2. Culture 2x500mL HYPERFlasks of double-synchronized parasites in supplemented RPMI and 2-4% hematocrit at 37°C, 5% O_2_, and 5% CO_2_.
3. Change media every 8-12 hours. The final media change prior to harvest should take place no more than 8, and no fewer than 4 hours preceding the harvest.
4. Harvest parasites when they have grown to 10-20% parasitemia, and are at the late trophozoite/early schizont stage; as late as possible in the life cycle prior to schizont segmentation.
5. Harvest by centrifuging at 1500rpm for 5 minutes at room temperature in tabletop centrifuge without brake.
6. Carefully remove media.
7. Resuspend pellet in ice-cold Buffer A, transfer to 50mL conical tube on ice
   1. Volume of Buffer A will vary depending on batch of saponin utilized; to be determined empirically as indicated in Additional File 1
8. Add ice-cold 0.15% saponin as quickly as possible, invert 2-3 times, return tubes to ice
   1. Volume of saponin to be added will vary depending on batch of saponin utilized; to be determined empirically as indicated in Additional File 1
9. Immediately centrifuge tubes at 10,000x*g* and 4^o^C for 10 minutes with low brake.
10. Keeping tubes on ice, carefully remove supernatant as quickly as possible.
11. Resuspend pellet in 45mL ice-cold Buffer A to wash.
12. Immediately centrifuge tubes at 10,000x*g* and 4^o^C for 10 minutes with low brake.
13. Keeping tubes on ice, carefully remove supernatant as quickly as possible.
14. Repeat wash: resuspend pellet in 45mL ice-cold Buffer A.
15. Immediately centrifuge tubes at 10,000x*g* and 4^o^C for 10 minutes with low brake.
16. On ice, carefully remove supernatant.
17. Estimate pellet volume, and resuspend pellet in an equal volume of Buffer B2, i.e. if pellet volume is 1mL, resuspend in 1mL Buffer B2
18. Transfer resuspended pellet to 1.5mL screw-top tube, flash freeze in liquid nitrogen
19. Store frozen pellets at -80^o^C until ready to homogenize.
20. Thaw pellet(s) on ice.
21. While thawing pellet(s), prepare homogenizer: assemble with 4μm-clearance ball bearing, pre-chill homogenizer on ice, flush homogenizer with 1.5mL Buffer B2, and remove all buffer. (Ball homogenizer from Isiobiotec).
22. Draw 1.5-3mL of thawed pellet into 3mL Luer lock syringe (pre-chilled on ice) and attach to prepared homogenizer.
23. Utilizing homogenizer robot, homogenize the thawed pellet by passing from syringe to syringe 20 times, generating lysate.
    1. If homogenizing pellets from more than one harvest, wash the homogenizer in between processing separate harvests with ice-cold Buffer B2.
24. Transfer homogenized lysate to pre-chilled 1.5mL microcentrifuge tube(s).
25. Centrifuge immediately at 16,000x*g* and 4^o^C for 10 minutes in tabletop microcentrifuge.
26. Carefully transfer supernatant (the PfIVT extract) to fresh pre-chilled tube, setting aside 100μL of extract in a separate tube for quality control testing.
27. Flash-freeze all tubes in liquid nitrogen, store at -80^o^C.
28. For each harvest, determine whether extract is sufficiently active for use (see Additional File 4 for flowchart):
    1. Thaw test aliquot, test in PfIVT assay (according to protocol in Additional File 5) with 2.0, 2.5, 3.0, 3.5, and 4.0 mM added magnesium (added in 10XTM), and 2 hour incubation time.
    2. Extracts achieving activity greater than or equal to 10^4^ RLU with firefly luciferase are considered good, those with lower activity are discarded.
29. Pool all extracts that surpass the activity threshold in a 50mL conical tube on ice. Aliquot into 200μL aliquots in pre-chilled tubes, flash freeze in liquid nitrogen.
30. Thaw two aliquots for activity testing (see Additional File 4 for flowchart):
    1. Test in PfIVT assay (according to protocol in Additional File 5) with 2.0, 2.5, 3.0, 3.5, and 4.0 mM added magnesium (added in 10XTM), and 90 minute incubation time. This should be done in duplicate. The magnesium concentration that yields the highest signal is the concentration that will be used for future assays with this pool of extract.
    2. Using the appropriate magnesium concentration as determined in step a, test in PfIVT assay to determine kinetics of the extract pool. Remove duplicate samples from 37C incubation every 15 minutes until the final time point of 150 minutes, placing on ice and adding 2μL of 50μM CHX STOP (see Additional File 5) to each sample immediately after removing from the incubator. For inhibitor assays, the incubation time used should be 70-80% of the activity at saturation, to maximize the assay window within the linear range. For different kinds of PfIVT assays, a shorter incubation may provide a better dynamic range.
