# Supplementary material for: The Plasmodium falciparum cytoplasmic translation apparatus: a promising therapeutic target not yet exploited by clinically approved anti-malarials
Source: Malar J. 2018 Dec 12;17:465. doi: 10.1186/s12936-018-2616-7 (PMC6292128; doi:10.1186/s12936-018-2616-7)
Supplement: Supplementary file 5 — Additional file 5. Method for PfIVT assay: step-by-step protocol. [file 12936_2018_2616_MOESM5_ESM.docx]

**Additional File 5. Method for PfIVT assay: step-by-step protocol.**

All reagents should be RNase-free. Proper RNA handling procedures must be followed to avoid introduction of RNase and subsequent degradation of mRNA transcripts and skewing of results.

**Solutions**

10X Translation Mix *DO NOT VORTEX 10XTM or creatine phosphokinase*

20mM HEPES pH7.4, 75mM KOAc, 2mM DTT, 5mM ATP, 1mM GTP, 200mM phosphocreatine, 2μg/μL creatine phosphokinase, appropriate 10X Mg(OAc)_2_ as determined for extract pool

*Aliquoted, flash-frozen in liquid nitrogen, stored at -80^o^C

Firefly luciferin reagent

Per 100mL: 89.98mL ddH_2_O; 0.20mL EDTA (50mM); 1.66mL MgSO_4_ (100mM); 3.33mL DTT (1M); 0.27mL CoA (100mM); 0.53mL ATP (100mM); 2mL tricine pH8.15 (1M); 1mL D-luciferin (100mM); 0.5mL NaOH (1M); 0.53mL magnesium carbonate hydroxide (50mM)

Aliquoted, flash-frozen in liquid nitrogen, stored at -80^o^C in the dark

Cycloheximide stop solution (CHX STOP) 50μM

Aliquoted, snap-frozen on dry ice, and stored at -20^o^C in the dark

1. Combine the following in PCR plates:

14μL PfIVT extract

2μL 10X Translation Mix with appropriate [Mg++]

1μL amino acid mixture (100μM)

1μL T7-transcribed firefly luciferase mRNA (1μg/μL stock)

2μL DEPC H_2_O

20μL total reaction volume

* For multiple reactions, create appropriate PfIVT reaction master mix and dispense to PCR plates

** If testing inhibitors or other drugs in the assay, pre-dispense to drug to PCR-plate and spin down. Then add PfIVT reaction to walls of PCR-plate wells and spin down such that all PfIVT reactions are mixed with drug at the same time.

*** Similarly, if testing magnesium concentrations, pre-dispense Mg(OAc)2 and DEPC H2O to PCR plates. Make magnesium-free PfIVT reaction master mix and dispense to walls of PCR-plate wells and spin down such that all PfIVT reactions are mixed with magnesium at the same time.

1. Pipette up and down gently to mix (DO NOT VORTEX)
2. If using PCR plates: fill empty wells with 20μL DEPC H_2_O and cover with adhesive plate seal to prevent evaporation
3. Spin down tubes/plates briefly
4. Incubate at 37oC for appropriate amount of time
5. Spin down tubes/plates briefly, place on ice
6. Dispense 2μL CHX STOP to appropriate wells of white 96-well flat-bottom assay plate, briefly spin down to ensure no liquid on walls of plate
7. CHX STOP plates can be prepared in advance and stored (with appropriate plate seals) for a limited time at -20^o^C, then thawed & brought to room temperature when needed.
8. After thawing, briefly spin down plates before use.
9. It is important that plates be brought to room temperature prior to addition of luciferin reagent, as luciferase enzyme activity and luminescence signal are temperature sensitive.
10. Carefully transfer 17.5μL of each PfIVT reaction to walls of wells containing CHX STOP (prepared in Step 7)
11. Spin down plates so that all PfIVT reactions mix with, and are stopped by, the CHX STOP at the same time.
12. Read plates at room temperature on injection luminometer with the following settings:
    - 200μL injection luciferin reagent
    - 200μL/sec injection rate
    - 3sec delay
    - 3sec integration
